# miR-190 Enhances HIF-Dependent Responses to Hypoxia in *Drosophila* by Inhibiting the Prolyl-4-hydroxylase Fatiga

**DOI:** 10.1371/journal.pgen.1006073

**Published:** 2016-05-25

**Authors:** Ana Laura De Lella Ezcurra, Agustina Paola Bertolin, Kevin Kim, Maximiliano Javier Katz, Lautaro Gándara, Tvisha Misra, Stefan Luschnig, Norbert Perrimon, Mariana Melani, Pablo Wappner

**Affiliations:** 1 Instituto Leloir, Buenos Aires, Argentina; 2 Department of Genetics, Harvard Medical School; Howard Hughes Medical Institute, Harvard Medical School, Boston, Massachusetts, United States of America; 3 Institute of Molecular Life Sciences, University of Zürich, Zürich, Switzerland; 4 Institute of Neurobiology, University of Münster; Cluster of Excellence EXC 1003, Cells in Motion, CiM, Münster, Germany; 5 Departamento de Fisiología y Biología Molecular, Facultad de Ciencias Exactas y Naturales, Universidad de Buenos Aires, Buenos Aires, Argentina; Stanford University School of Medicine, UNITED STATES

## Abstract

Cellular and systemic responses to low oxygen levels are principally mediated by Hypoxia Inducible Factors (HIFs), a family of evolutionary conserved heterodimeric transcription factors, whose alpha- and beta-subunits belong to the bHLH-PAS family. In normoxia, HIFα is hydroxylated by specific prolyl-4-hydroxylases, targeting it for proteasomal degradation, while in hypoxia the activity of these hydroxylases decreases due to low oxygen availability, leading to HIFα accumulation and expression of HIF target genes. To identify microRNAs required for maximal HIF activity, we conducted an overexpression screen in *Drosophila melanogaster*, evaluating the induction of a HIF transcriptional reporter. miR-190 overexpression enhanced HIF-dependent biological responses, including terminal sprouting of the tracheal system, while in miR-190 loss of function embryos the hypoxic response was impaired. In hypoxic conditions, miR-190 expression was upregulated and required for induction of HIF target genes by directly inhibiting the HIF prolyl-4-hydroxylase Fatiga. Thus, miR-190 is a novel regulator of the hypoxia response that represses the oxygen sensor Fatiga, leading to HIFα stabilization and enhancement of hypoxic responses.

## Author Summary

Sufficient oxygen supply is essential for animal survival. When cells or organisms are exposed to low oxygen levels (hypoxia), a complex molecular response is triggered, enabling adaptation to this stressful condition. A key mediator of this response is HIF, a transcription factor that induces the expression of a set of genes that mediate the adaptive response to hypoxia. The most important regulation of HIF is exerted by a family of prolyl-4-hydroxylases (PHDs), which prevent HIF accumulation under normal oxygen levels and lift this inhibition of HIF only in hypoxia. This pathway is highly conserved among metazoans, including humans and the fruit fly *Drosophila melanogaster*. microRNAs (miRNAs), which are small (~22 nucleotides long), non-coding RNAs that control gene expression post-transcriptionally, play central roles in stress responses. In the present study, we have performed a screen in *Drosophila* and identified miRNAs that regulate HIF-dependent adaptations to hypoxia. We found one miRNA, miR-190, that is induced in hypoxia and in turn enhances HIF-dependent biological responses, as well as the expression of HIF-inducible genes. The mechanism of action of miR-190 involves the inhibition of the *Drosophila* PHD, thereby positively regulating HIF-dependent responses to hypoxia at the molecular and organismal level.

## Introduction

Cells and organisms exposed to environmental stress mount complex adaptive responses in order to maintain homeostasis. In mammals, hypoxic stress triggers cellular and systemic modifications, such as metabolic switches [1,2], erythropoiesis [3,4], angiogenesis and vasodilation [5,6], resulting in reduced oxygen consumption and increased oxygen transport to hypoxic tissues. Responses to hypoxia are principally mediated by a family of transcription factors named Hypoxia Inducible Factors (HIFs) [7–12], that are heterodimers composed of an oxygen regulated α-subunit (HIFα) and a constitutive β-subunit (HIFβ) [13,14]. HIFα activity is controlled by different mechanisms [15], the most prevalent being oxygen-dependent regulation of protein stability. In normoxia, HIFα is hydroxylated on two specific prolyl residues within the oxygen-dependent degradation (ODD) domain, enabling binding to the von Hippel-Lindau (VHL) tumor suppressor protein, a component of the elongin BC/cullin-2/VHL ubiquitin-protein ligase complex, which targets HIFα for degradation at the 26S proteasome [16–18]. HIFα hydroxylation is catalyzed by specific prolyl-4-hydroxylases (PHD1-PHD3) that are 2-oxoglutarate and Fe(II)-dependent dioxygenases [19,20]. Since PHDs use molecular oxygen as a co-substrate of the reaction, in hypoxia their activity is inhibited. Consequently, in hypoxia HIFα is not hydroxylated, accumulates, translocates to the nucleus, dimerizes with HIFβ and binds to HIF-responsive elements (HREs), thus promoting transcription of target genes [21–23].

We and others have demonstrated that *Drosophila melanogaster* has a hypoxia-inducible transcriptional response that is homologous to that of mammals [24], with Similar (Sima) [25] and Tango (Tgo) [26] being the homologs of HIFα and HIFβ, respectively [27,28], and Fatiga (Fga) the only *Drosophila* PHD enzyme [29,30].

We have previously shown that the microRNA (miRNA) machinery is required for full activation of the Sima-dependent transcriptional response to hypoxia, both in cell culture and *in vivo* [31]. Yet, the individual miRNAs involved in Sima regulation remained unrevealed. Here, we performed an overexpression screen in *Drosophila* embryos aimed at defining miRNAs that regulate the hypoxic response, and identified specific miRNAs whose overexpression enhances Sima-dependent transcription. One of these miRNAs, miR-190, is induced in hypoxia, is necessary for Sima-dependent gene expression and promotes terminal tracheal cell sprouting. Finally, we found that miR-190 directly targets the HIF prolyl hydroxylase *fatiga* transcript on its 3’UTR, thereby inhibiting its expression. We propose that miR-190 positively regulates Sima-dependent transcription by inhibiting the oxygen sensor Fatiga, which is the main negative regulator of the hypoxic response.

## Results

### Screen to identify miRNAs that regulate the transcriptional response to hypoxia in *Drosophila*

**Fig 1:**
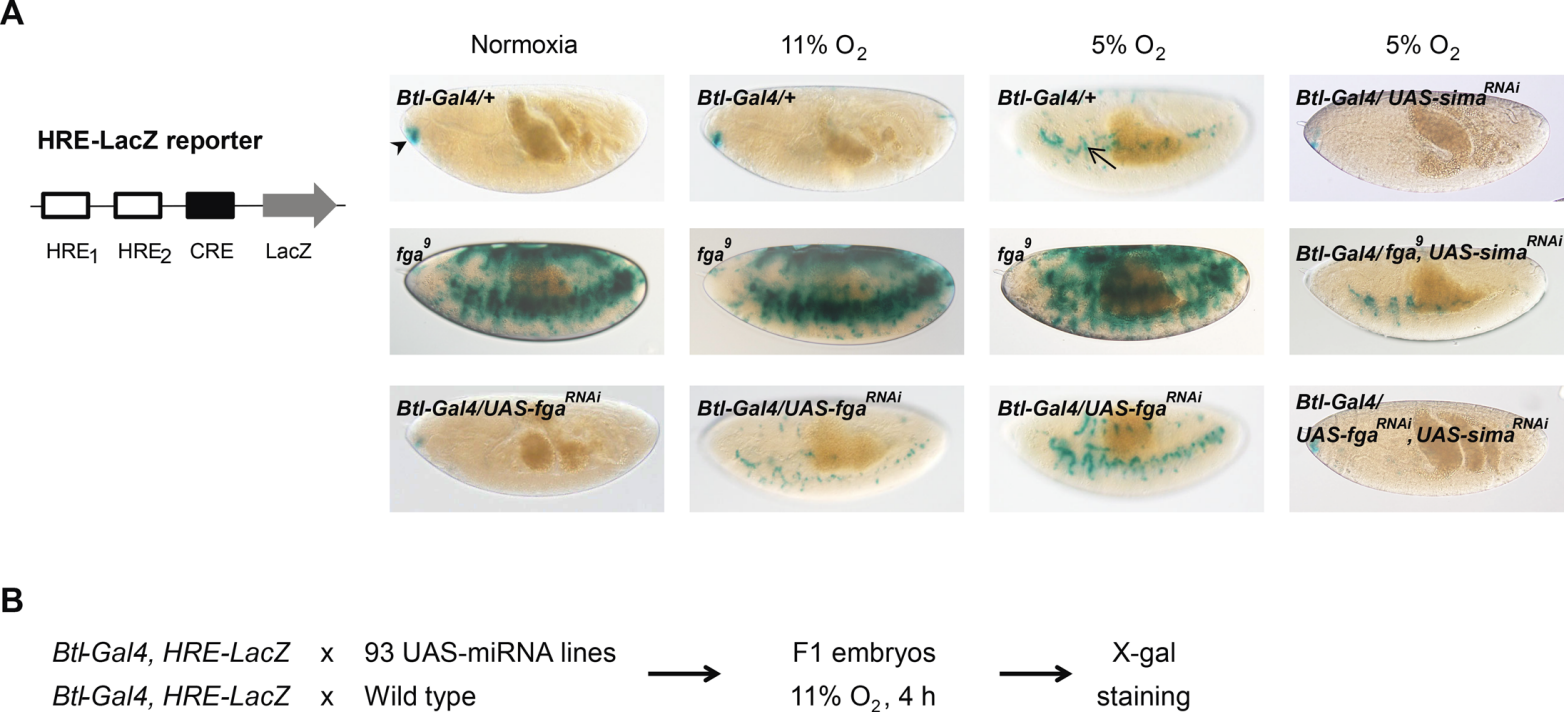
Screen for miRNAs that enhance the hypoxic response. **(A)** Left: Schematic representation of the HRE-LacZ reporter. The enhancer derived from the murine *lactate dehydrogenase-A* (*ldh-A*), which contains two HIF-responsive elements (HREs) and one cyclic AMP-responsive element (CRE), controls the expression of β-galactosidase in a HIF/Sima-dependent manner. Right: The reporter was not expressed in embryos with the *btl*-Gal4 driver alone in normoxia or mild hypoxia (11% O_2_), and was induced in these embryos exposed to strong hypoxia (5% O_2_, arrow); hypoxic induction of the reporter was suppressed by the expression of *sima* RNAi. In embryos homozygous for the *fga*^*9*^ mutant allele, the HRE-LacZ reporter was strongly induced even in normoxia, but this induction was largely reduced by simultaneous expression of *sima* RNAi. Expression of a *fatiga* RNAi, which has a modest effect, was not sufficient to induce reporter expression in normoxia, but provoked induction at 11% O_2_ and enhancement of the response at 5% O_2_. Reporter expression in these conditions was also suppressed by coexpression of *sima* RNAi. The staining observed at the anterior tip of the embryo (arrowhead) is hypoxia-independent. **(B)** Design of the screen. A collection of 93 miRNAs was overexpressed with *btl*-Gal4 in stage 14–17 embryos that also bear the HRE-LacZ reporter. The negative control expressed a *btl*-Gal4 driver along with the HRE-LacZ reporter, but not a miRNA. Embryos were exposed to 11% O_2_ for 4 h, after which X-gal staining was performed.

To identify miRNAs involved in the response to hypoxia in *Drosophila*, we performed an overexpression screen in stage 14–17 embryos. The rationale was that since suppression of the miRNA machinery inhibits the hypoxic response [31], overexpression of certain specific miRNAs could potentially enhance this response. For the screen, we utilized a collection of 93 fly lines (S1 Table) to overexpress individual miRNAs under control of a *breathless*-Gal4 (*btl*-Gal4) driver, and a HIF/Sima-dependent LacZ reporter (HRE-LacZ reporter) as a read out (Fig 1A; [27]). This transgenic reporter was not expressed in normoxic embryos, but induced at 5% O_2_ in a Sima-dependent manner (Fig 1A; [27]). In *fatiga* homozygous mutant embryos (*fga*^*9*^), Sima protein accumulates [29], and hence, expression of the reporter was strongly upregulated even in normoxia [29], being this induction suppressed by expression of *sima* RNAi (Fig 1A and S1 Fig). Given that the biological effect of *Drosophila* miRNAs is often mild, we sought to conduct the screen under sensitized conditions. To define an appropriate sensitized condition of the hypoxia response system, we used a *UAS-fatiga RNAi* line (*fatiga*^RNAi^; [32]) whose effect is modest. In normoxia, expression of *fatiga*^RNAi^ had no effect on HRE-LacZ reporter induction, while at mild hypoxia (11% O_2_), β-galactosidase expression was readily detectable in these embryos (Fig 1A). In embryos bearing only the *btl*-Gal4 driver, no induction of the reporter was observed under these same conditions (Fig 1A). In strong hypoxia (5% O_2_), reporter expression was enhanced in the *fatiga*^RNAi^ line in comparison to wild type controls (Fig 1A). Since mild hypoxia (11% O_2_) represented a sensitized condition for the hypoxia response machinery, in which potential effects of miRNAs regulating the system might become evident, we performed the screen by exposing the embryos that overexpressed miRNAs at 11% O_2_ for 4 h; isogenic embryos that did not overexpress any miRNA were used as negative controls (Fig 1B).

**Fig 2:**
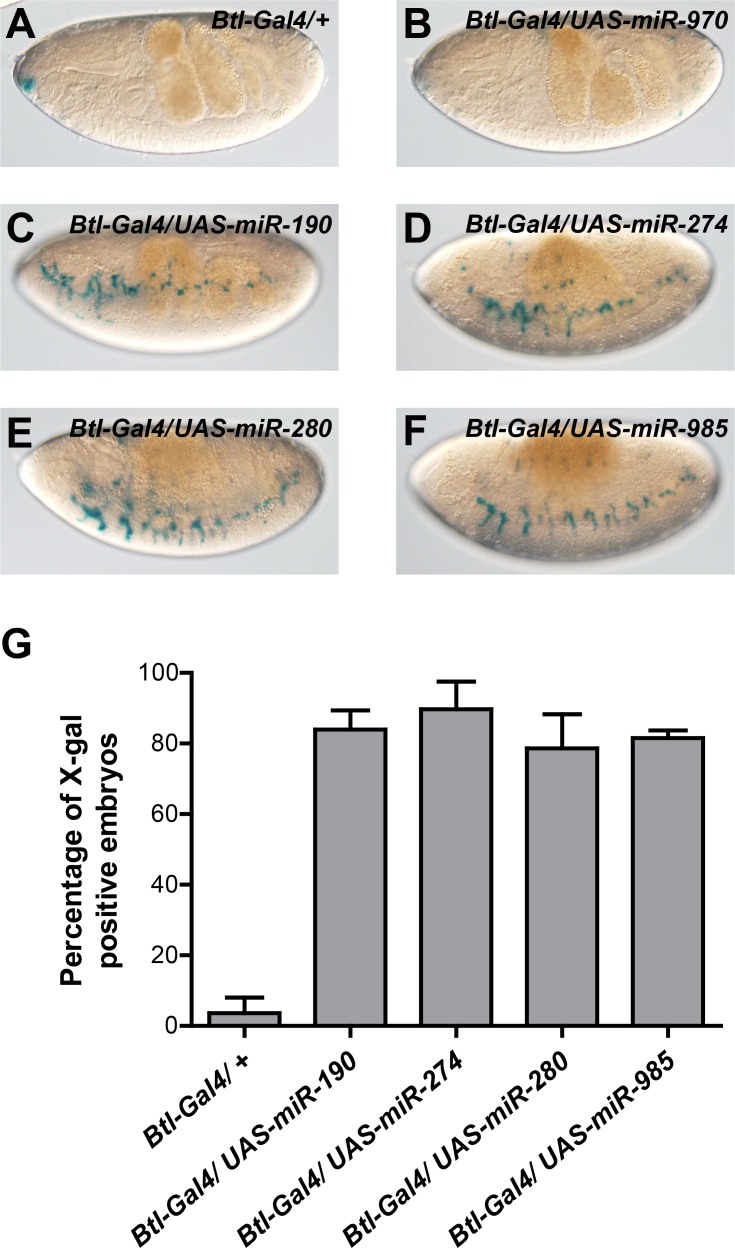
A specific set of miRNAs enhances the hypoxic response. miRNAs were overexpressed using a *btl*-Gal4 driver in embryos exposed to 11% O_2_. **(A)** No induction of the reporter was observed in embryos with the *btl*-Gal4 driver alone (negative control) and **(B)** in embryos overexpressing most of the microRNAs of the collection (miR-970 is shown as an example). **(C)** Expression of miR-190, **(D)** miR-274, **(E)** miR-280 or **(F)** miR-985 induced the Sima-dependent HRE-LacZ reporter. **(G)** Quantification of embryos in which the HRE-LacZ reporter was induced. Error bars represent SD; n ≥ 25 per group in three independent experiments.

The screen was carried out in triplicate; overexpression of most miRNAs had no effect on HRE-LacZ reporter expression (Fig 2A and 2B), but 4 out of the 93 tested miRNAs, namely miR-190 (Fig 2C and 2G), miR-274 (Fig 2D and 2G), miR-280 (Fig 2E and 2G) and miR-985 (Fig 2F and 2G), scored as positives in the screen, inducing expression of the reporter. miR-970, one of the many miRNAs that had no effect on reporter expression, was randomly chosen as a negative miRNA control, and used in the rest of the experiments carried out in this work. We focused our studies on miR-190, whose occurrence *in vivo* has been experimentally validated by high-throughput sequencing of small RNA libraries generated from different tissues and developmental stages [33,34].

### miR-190 overexpression induces lethality and an increase of tracheal terminal sprouting in a Sima-dependent manner

In order to confirm miR-190 participation in the Fatiga/Sima pathway, we began by studying biological responses characteristic of Sima accumulation. We previously reported that *fatiga* loss-of-function mutations provoke accumulation of high levels of Sima in normoxia, resulting in lethality at the pupal stage [29] and an increased number of terminal ramifications in 3^rd^ instar larval tracheae [35]. Since our results suggested that miR-190 is a positive regulator of Sima (Fig 2), we tested whether overexpression of miR-190 can also induce similar developmental phenotypes, and to what extent they depend on Sima activity.

**Fig 3:**
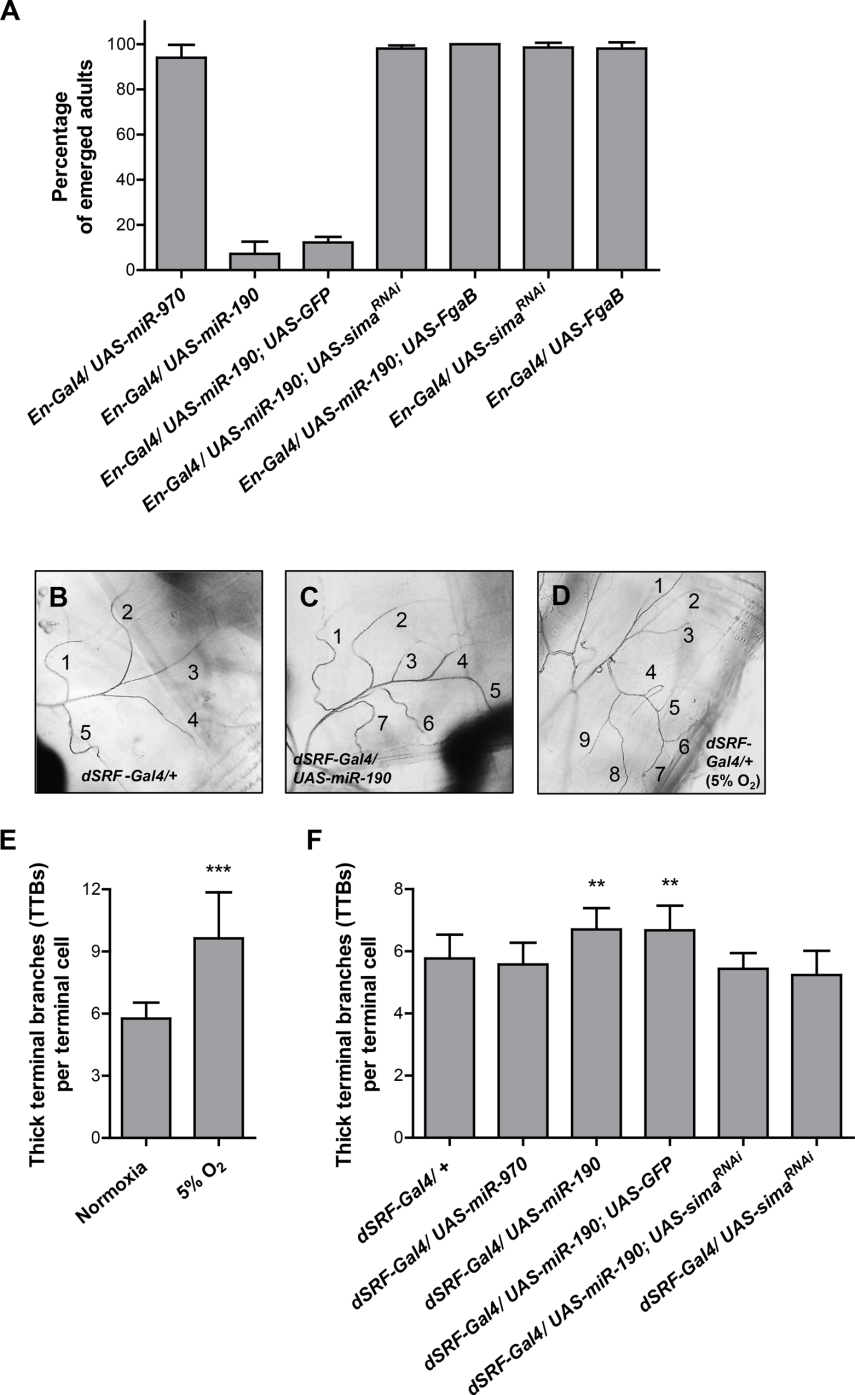
miR-190 overexpression causes lethality and enhances tracheal terminal branching in a Sima-dependent manner. **(A)** Different UAS transgenes were expressed under control of an *en*-Gal4 driver. Overexpression of miR-190 (*en-Gal4/UAS-miR-190* and *en-Gal4/UAS-miR-190; UAS-GFP*) provoked pupal lethality. Sima knock-down (*en-Gal4/UAS-miR-190; UAS-sima*^*RNAi*^), or overexpression of the isoform B of Fatiga (*en-Gal4/UAS-miR-190; UAS-FgaB*), rescued viability, as assessed by emergence of the adults from the puparium. Individuals expressing only *sima* RNAi or overexpressing Fatiga B did not show alterations in viability. Error bars represent SD; n ≥ 25 per group. **(B-F)** Expression of different constructs in tracheal terminal cells was achieved using a *dSRF*-Gal4 driver. **(B-C)** Overexpression of miR-190 enhanced tracheal branching of 3^rd^ instar larvae maintained in normoxia, as compared to normoxic larvae bearing the driver alone. **(D)** Exposure of larvae to hypoxia (5% O_2_) during 48 h strongly enhanced the number of tracheal terminal branches. **(E-F)** Quantification of terminal ramifications. The number of terminal cell projections was quantified as previously reported [35]. (E) The number of thick terminal branches of control larvae carrying the *dSRF*-Gal4 driver significantly increased when they were exposed to hypoxia (5% O_2_ for 48 h). ***p<0.001; unpaired two-tailed Student’s *t*-test with Welch's correction. Error bars represent SD; n ≥ 20 per group. (F) Overexpression of miR-190 provoked a significant enhancement of ramification in normoxic 3^rd^ instar larvae, which was suppressed by co-expression of *sima* RNAi. **p<0.01; Kruskal-Wallis one-way ANOVA. Error bars represent SD; n ≥ 15 per group.

When overexpressed with an *engrailed*-Gal4 (*en*-Gal4) driver, miR-190, but not the control miRNA (miR-970), was associated with lethality at pupal or pharate adult stages (Fig 3A). Knock-down of *sima* by RNAi completely rescued the lethality caused by miR-190 overexpression, suggesting that lethality was indeed due to Sima accumulation (Fig 3A). In addition, coexpression of Fatiga B, one of the isoforms of the *Drosophila* HIF prolyl hydroxylase, also rescued the lethal phenotype (Fig 3A), further suggesting that over-accumulation of Sima was the causal factor. When expressed alone, neither *sima* RNAi nor Fatiga B overexpression had effects on viability (Fig 3A).

Tracheal terminal cells of *Drosophila* 3^rd^ instar larvae are plastic and ramify in response to hypoxia (Fig 3D and 3E; [36]) in a Sima- and Fatiga-dependent manner [35,37]. As we previously reported, the number of terminal branches with more than 1 μm diameter (“thick terminal branches”, TTBs) of the dorsal branch of the 3^rd^ segment of 3^rd^ instar larvae is a sensitive parameter to quantify terminal tracheal branching after physiological or genetic interventions [35]. To investigate whether miR-190 can also modulate this process, we overexpressed miR-190 under control of the tracheal terminal cell-specific driver *dSRF*-Gal4. In normoxic larvae overexpressing this miRNA, we observed a significant increase in the number of TTBs (Fig 3C and 3F) in comparison to controls expressing the Gal4 driver only (Fig 3B and 3F), or larvae overexpressing an unrelated miRNA (miR-970) (Fig 3F). To investigate if this increase of ramification depends on Sima, we coexpressed miR-190 along with a UAS-*sima*^RNAi^, and observed complete reversion of the phenotype, attaining these larvae a normal number of TTBs (Fig 3F). Expression of the *sima* RNAi on itself did not induce changes in tracheal terminal sprouting. These results indicate that overexpression of miR-190 can induce Sima-dependent tracheal terminal sprouting, a typical physiological response to hypoxia.

### miR-190 enhances Sima-dependent transcription

**Fig 4:**
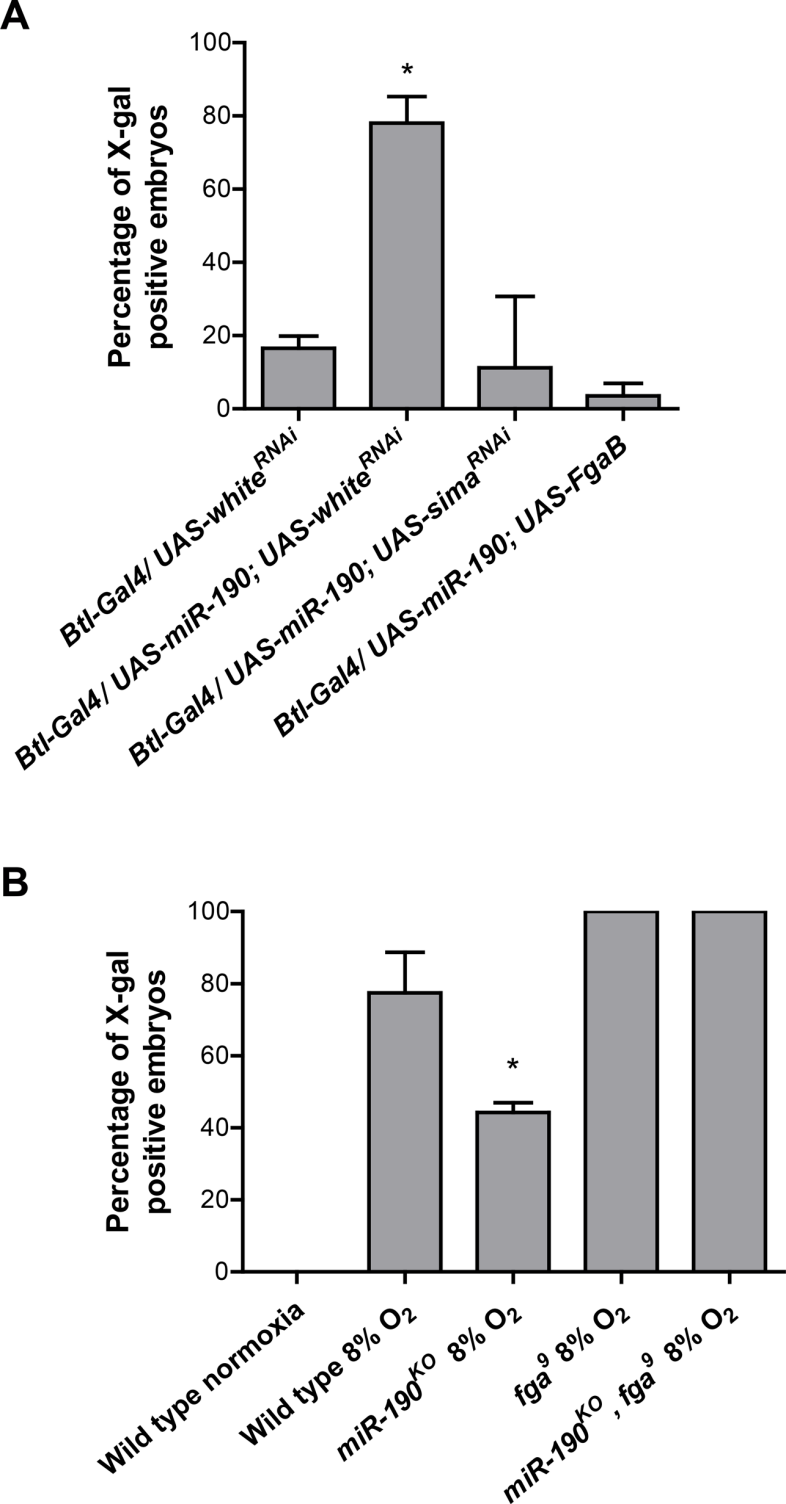
miR-190 induces the expression of the HRE-LacZ reporter in a Sima-dependent manner. Embryos were maintained in normoxia or exposed to mild hypoxia (8% O_2_ for 4 h) and, after X-gal staining, the percentage of embryos expressing the reporter was quantified. A *btl*-Gal4 driver was used to induce different constructs in the tracheal system. **(A)** Overexpression of miR-190 (*btl-Gal4/UAS-miR-190; UAS-white*^*RNAi*^) induced the HRE-LacZ reporter, and induction was reduced by coexpression of a *sima* RNAi (*btl-Gal4/UAS-miR-190; UAS-sima*^*RNAi*^) or overexpression of Fatiga B (*btl-Gal4/ UAS-miR-190; UAS-FgaB*). *p<0.05; one-way ANOVA, followed by Fisher's least significant difference (LSD) *post hoc* test. Error bars represent SD; n ≥ 30 per group in three independent experiments. **(B)** In wild type embryos carrying two copies of the HRE-LacZ reporter, induction could be observed already at 8% O_2_, and this induction was significantly reduced in *miR-190*^*KO*^ homozygous embryos. *fatiga* homozygous mutants (*fga*^*9*^) exhibited induction of the reporter, which did not decrease in double homozygous *miR-190*^*KO*^, *fga*^*9*^ mutants, suggesting that miR-190 operates upstream to Fatiga. *p<0.05; one-way ANOVA, followed by Fisher's least significant difference (LSD) *post hoc* test (data were transformed using natural logarithm to fulfill variance homogeneity criteria). Error bars represent SD; n ≥ 10 per group in three independent experiments.

To get additional evidence that miR-190 participates in the HIF pathway, we analyzed genetic interactions between miR-190, *fatiga* and *sima*, by assessing induction of the HRE-LacZ reporter as a read out. Overexpression of miR-190 with a *btl*-Gal4 driver in mild hypoxia enhanced expression of the HRE-LacZ reporter (Fig 2) in comparison with control individuals expressing an unrelated RNAi (Fig 4A); co-expression of this miRNA along with *sima* RNAi suppressed this enhancement (Fig 4A). Overexpression of miR-190 along with Fatiga B, a highly active isoform of the oxygen sensor Fatiga [30], sharply decreased induction of the reporter (Fig 4A). These results indicate that miR-190 enhances the HIF pathway, antagonizing the activity of the prolyl-4-hydroxylase Fatiga.

To analyze further these genetic interactions, we utilized miR-190 null mutant embryos (*miR-190*^*KO*^, [38]). Unlike the previous experiments in which the HRE-LacZ reporter was utilized in heterozygosis (Figs 1, 2 and 4A), the reporter was used in homozygosis to favor reporter induction in wild type embryos exposed to mild hypoxia (Fig 4B). Noteworthy, this induction was suppressed in *miR-190*^*KO*^ mutants (Fig 4B), confirming that miR-190 contributes to Sima-dependent transcription. In *fatiga* homozygous mutant embryos (*fga*^*9*^), induction of the reporter occurs (Figs 1A and 4B; [29]), and interestingly, this expression was not altered in *miR-190*^*KO*^ homozygotes (Fig 4B), indicating that miR-190 operates upstream of the *fatiga* gene. Taken together, our genetic interactions data are consistent with a model in which miR-190 inhibits Fatiga, resulting in an enhancement of the hypoxic response.

### miR-190 enhances transcription of endogenous Sima target genes

Having analyzed HRE-LacZ reporter induction upon miR-190 loss- and gain-of-function, we studied if miR-190 affects the expression of endogenous Sima target genes. We measured mRNA levels of two well-established Sima targets by real time RT-PCR, namely *fatiga B* (*fgaB*) and *heat shock factor* (*hsf*) [30,39] in embryos with gain- or loss-of-function of miR-190.

**Fig 5:**
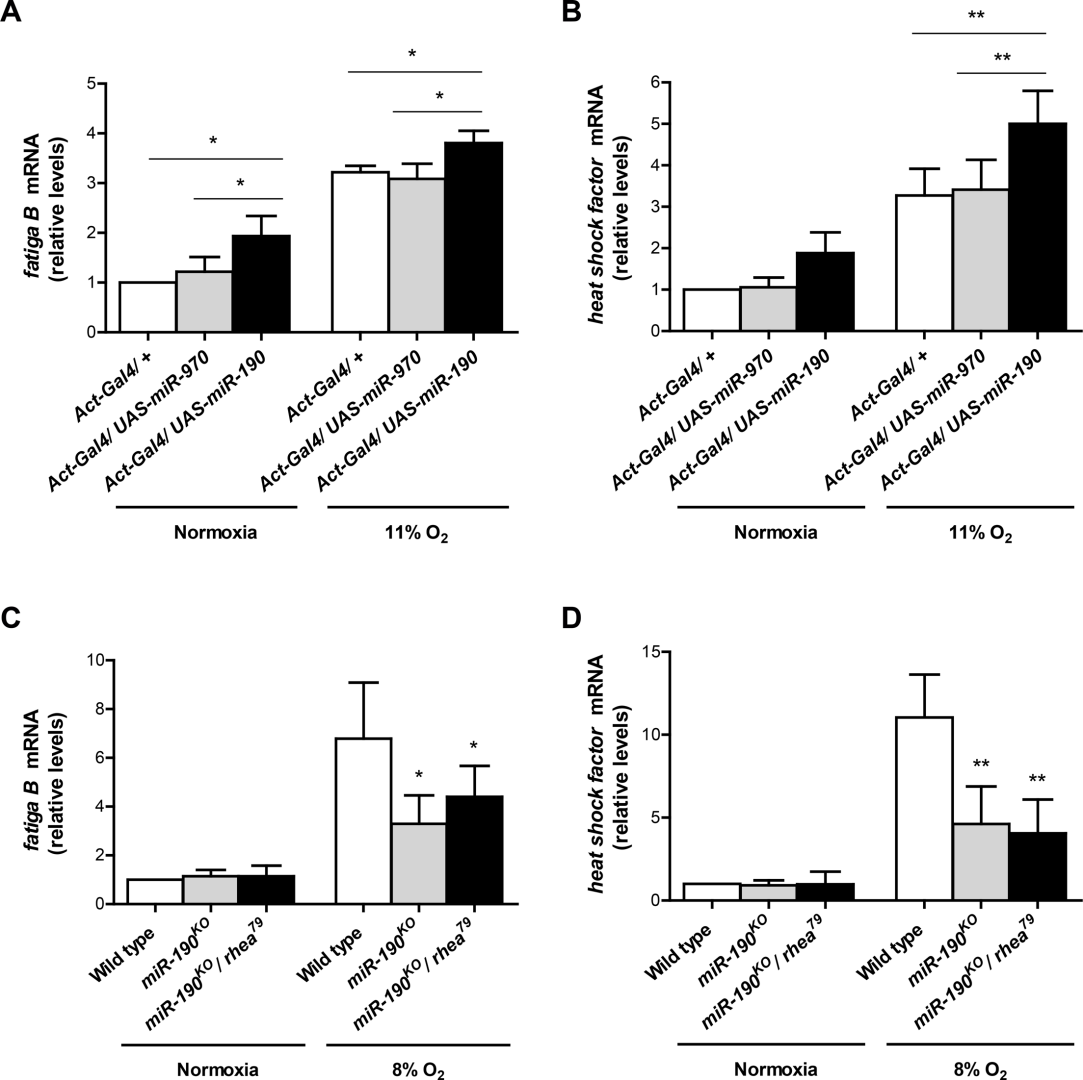
miR-190 enhances induction of Sima endogenous target genes. Transcript levels of two endogenous Sima target genes, *fatiga B* (*fgaB*) and *heat shock factor* (*hsf*), were analyzed by real time RT-PCR following overexpression of miR-190 or in *miR-190*^*KO*^ embryos. Embryos were either kept in normoxia or exposed to mild hypoxia (8–11% O_2_) during 4 h. **(A-B)** miR-190 or miR-970 (negative control) were overexpressed ubiquitously using an *act*-Gal4 driver. (A) Both in normoxia and mild hypoxia, overexpression of miR-190 enhanced *fgaB* mRNA levels, as compared to control embryos bearing the *act*-Gal4 driver alone or overexpressing miR-970 as a negative control. (B) *hsf* transcript levels were increased in embryos overexpressing miR-190 in mild hypoxia. *p<0.05, **p<0.01; two-way ANOVA, followed by Fisher's least significant difference (LSD) *post hoc* test. Error bars represent SD; n ≥ 3 per group. **(C-D)** In mild hypoxia, expression of the Sima target genes (*fgaB* and *hsf)* was reduced in *miR-190*^*KO*^ homozygous embryos, or in embryos heterozygous for *miR-190*^*KO*^ and the *rhea*^*79a*^ microdeletion. *p<0.05, **p<0.01; two-way ANOVA, followed by Fisher's least significant difference (LSD) *post hoc* test. Error bars represent SD; n ≥ 3 per group.

Ubiquitous overexpression of miR-190 with an *actin*-Gal4 (*act*-Gal4) driver in embryos maintained in normoxia or exposed to mild hypoxia (11% O_2_) for 4 h induced upregulation of *fgaB* and *hsf* transcripts in comparison to control embryos carrying only the *act*-Gal4 driver or overexpressing a control miRNA (Fig 5A and 5B). We confirmed these results in *Drosophila* S2R+ cells, where overexpression of miR-190 also resulted in upregulation of both *fgaB* and *hsf* mRNAs, in comparison with cells transfected with the empty vector (S2 Fig).

Next, we examined whether hypoxic induction of the HIF target genes *fgaB* and *hsf* is affected in miR-190 knock-out (*miR-190*^*KO*^) homozygous embryos or in embryos heterozygous for *miR-190*^*KO*^ and the *rhea*^*79a*^ microdeletion that covers the *rhea* locus [40]; miR-190 is encoded in an intron of the *rhea* gene [33,34] (S3 Fig). Hypoxic induction of both HIF target genes was severely impaired in miR-190 loss-of-function embryos (Fig 5C and 5D), indicating that miR-190 is necessary for HIF activation.

### miR-190 directly targets and downregulates the prolyl hydroxylase *fatiga*

The results described so far demonstrate that miR-190 positively regulates Sima. Therefore, to investigate the mechanisms of Sima regulation by miR-190, we measured *sima* mRNA abundance following miR-190 overexpression. Using a ubiquitous *act*-Gal4 driver, we overexpressed miR-190 in embryos exposed to either normoxia or mild hypoxia (11% O_2_) for 4 h, and measured *sima* mRNA levels by quantitative real time RT-PCR. No differences were detectable in *sima* transcript levels, either in normoxia or in mild hypoxia (S4 Fig), indicating that the miR-190 regulatory mechanism is independent of *sima* transcription or mRNA stability.

**Fig 6:**
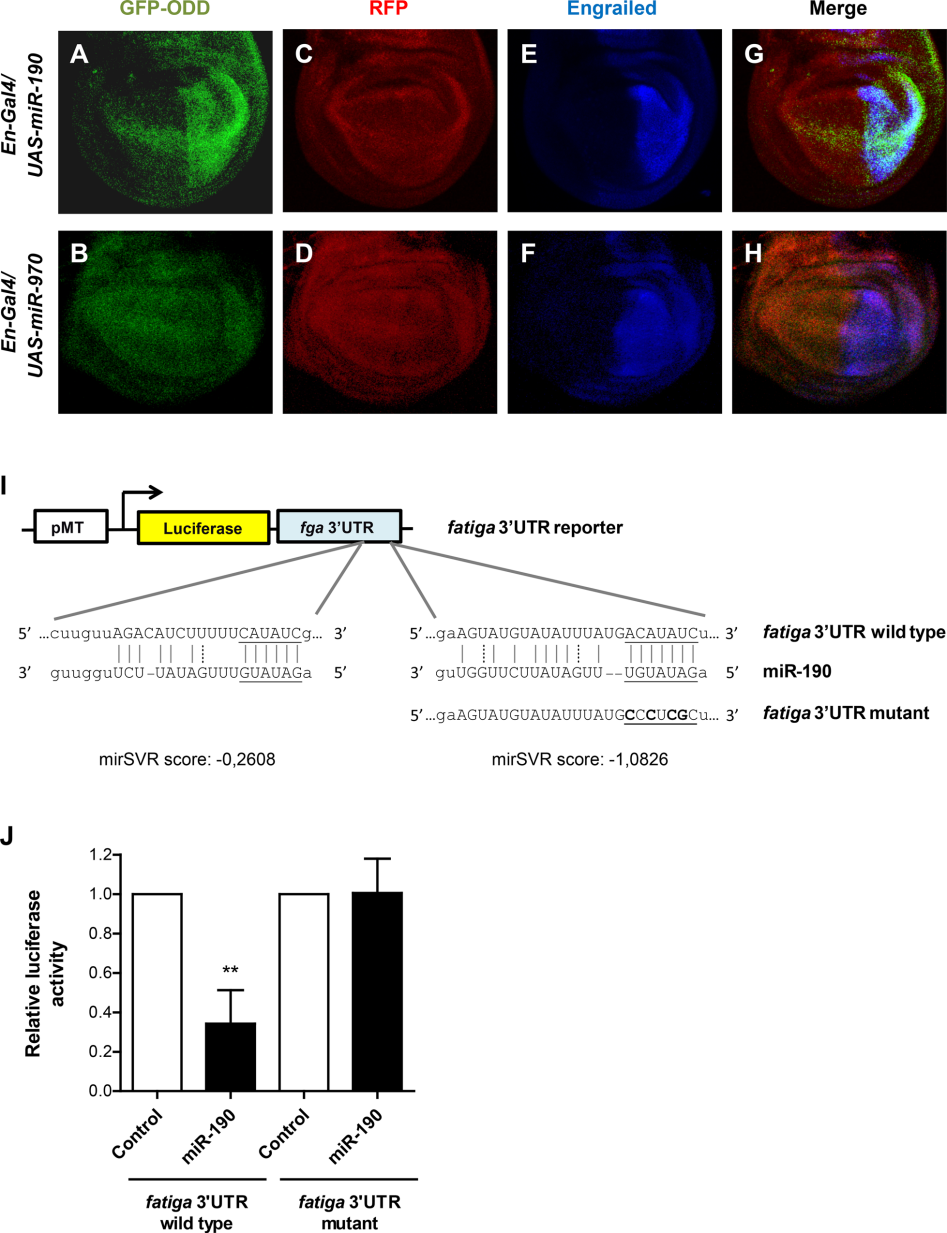
*fatiga* is a miR-190 direct target. **(A-H)** miR-190 reduces Fatiga activity. miR-190 or miR-970 (negative control) were overexpressed with an *en*-Gal4 driver in the wing disc posterior compartment of a line carrying the Fatiga reporter element GFP-ODD. (A) Overexpression of miR-190 provoked stabilization of the GFP-ODD construct in the posterior compartment of the imaginal disc, (B) while expression of miR-970 (negative control) did not affect GFP-ODD reporter behavior. (C-D) RFP protein expression shows that the reporter was expressed homogeneously throughout the disc. (E-F) Anti-Engrailed staining marks the posterior compartment of the disc. n ≥ 10 per group. **(I-J)** miR-190 directly targets the prolyl hydroxylase *fatiga*. (I) Schematic representation of *fatiga* 3’UTR reporter, in which the firefly luciferase coding sequence is fused to *fatiga* 3’UTR. Sequence alignment between miR-190 and the two predicted recognition sites at the 3’UTR of *fatiga* are shown. Lines indicate canonical pairings and dots indicate non-canonical (G:U) pairings; the seed regions are underlined. *fatiga* 3’UTR was mutated in four nucleotides of the seed region (bold) of the strongest recognition site. (J) *Drosophila* S2R+ cells were co-transfected with the *fatiga* 3’UTR reporter along with a pAc-miR-190 overexpression plasmid, or the empty vector as a control. In all cases, plasmids were co-transfected with a pMT-Renilla luciferase plasmid for normalization. Overexpression of miR-190 inhibited *fatiga* luciferase reporter expression, but had no effect on the reporter in which the 3’UTR of *fatiga* was mutagenized. **p<0.01; two-way ANOVA, followed by Fisher's least significant difference (LSD) *post hoc* test. Error bars represent SD; n ≥ 3 per group.

To identify direct targets of miR-190, we searched for target genes related to HIF-dependent response to hypoxia using publicly available database. The miRNA target prediction database miRanda (www.microrna.org) [41–43] predicted two potential miR-190 binding sites within the 3’ UTR of the prolyl-4-hydroxylase *fatiga*, the main negative regulator of Sima. To determine whether miR-190 can regulate *fatiga* expression, we used a transgenic reporter construct that directly responds to Fatiga activity. This ubiquitously expressed reporter construct consists of a Green Fluorescent Protein (GFP) fused to the Sima oxygen-dependent degradation (ODD) domain, which is rapidly degraded when Fatiga is active. Conversely, the fusion protein accumulates when Fatiga activity diminishes (Tvisha Misra and Stefan Luschnig, personal communication). We overexpressed miR-190 or a control miRNA with an *engrailed*-Gal4 driver in the posterior compartment of wing imaginal discs, and analyzed the behavior of the GFP-ODD reporter by confocal microscopy. While expression of the control miRNA (miR-970) did not induce changes in GFP-ODD reporter levels, expression of miR-190 resulted in increased GFP signal in the posterior compartment of the discs (Fig 6A–6H), indicating a stabilization of the GFP-ODD reporter, and suggesting downregulation of Fatiga. A Red Fluorescent Protein (RFP) expressed under the same ubiquitous promoter was used as an expression reference construct. RFP labeling was homogenous throughout the disc and therefore unaffected by expression of the miRNAs (Fig 6C and 6D).

To investigate whether *fatiga* is a direct target of miR-190, we analyzed the expression of a luciferase reporter in which the firefly luciferase coding sequence is fused to the 3’UTR of *fatiga* (Fig 6I). The experiment was carried out in S2R+ cells transfected with a plasmid driving the expression of miR-190, in comparison to cells transfected with an empty vector; miR-12 and its specific luciferase reporter [31,44] were utilized as a positive control of the system (S5 Fig). Importantly, transfection of the plasmid expressing miR-190 strongly reduced luciferase activity of the reporter containing the *fatiga* 3’UTR, as compared to control cells transfected with the empty vector (Fig 6J). To assess binding specificity of miR-190, we mutagenized the strongest miR-190 recognition site within the *fatiga* 3’UTR (Fig 6I). The reporter bearing the mutant binding site became insensitive to the expression of miR-190 (Fig 6J), confirming specificity of the miRNA. Collectively, these data demonstrate that miR-190 directly targets and downregulates *fatiga*.

### miR-190 is induced in hypoxia in a Sima-independent manner

**Fig 7:**
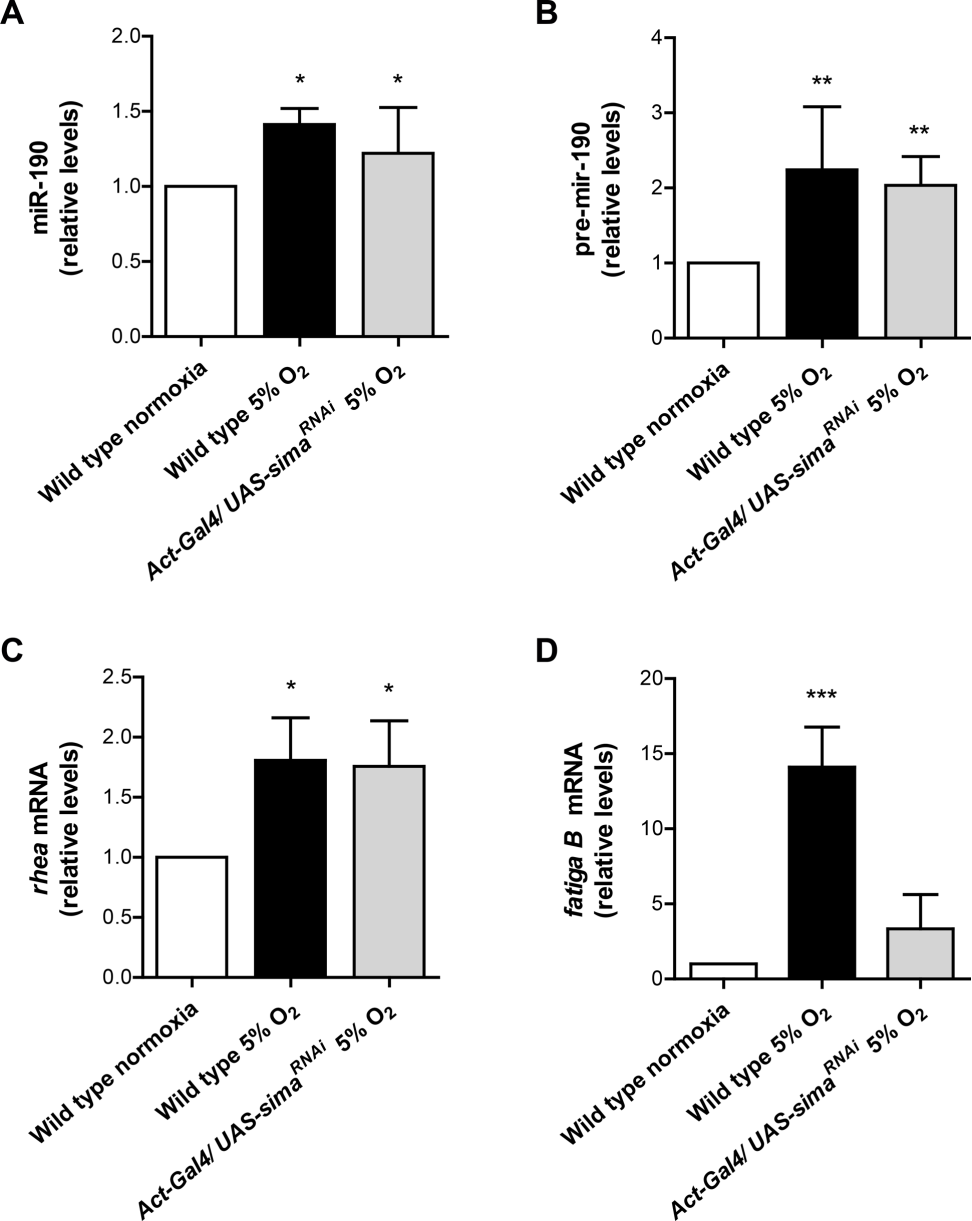
Hypoxic induction of miR-190. Embryos were exposed to 5% O_2_ for 4 h or maintained in normoxia, and expression of **(A)** miR-190, **(B)** pre-miR-190, **(C)** *rhea* and **(D)** *fgaB* transcript levels were assessed by real time RT-PCR. miR-190, pre-miR-190 and *rhea* were induced in hypoxia in a Sima-independent manner. The *fgaB* transcript, used as a positive control for Sima-dependent regulation, was strongly induced in hypoxia, and induction was reduced in embryos expressing *sim*a RNAi. *p<0.05, **p<0.01, ***p<0.001; one-way ANOVA, followed by Fisher's least significant difference (LSD) *post hoc* test (in B data were transformed using the reciprocal number to fulfill variance homogeneity criteria). Error bars represent SD; n ≥ 3 per group.

We next investigated if miR-190 expression is regulated by oxygen. RT-qPCR analysis revealed a significant increase of miR-190 expression in wild type embryos exposed to hypoxia (5% O_2_ for 4 h), in comparison to controls maintained in normoxia (Fig 7A). To determine if hypoxic induction of miR-190 depends on Sima, we analyzed miR-190 levels in embryos exposed to hypoxia and expressing *sima* RNAi. *sima* knock-down did not affect miR-190 hypoxic induction, suggesting that upregulation of miR-190 in hypoxia is independent of Sima (Fig 7A).

To investigate if miR-190 upregulation in hypoxia is regulated at a transcriptional level, we evaluated the expression of pre-miR-190. As depicted in Fig 7B, pre-miR-190 expression increased in hypoxia as compared to normoxia, and this induction was again unaffected after *sima* knock-down. These results suggest that hypoxic upregulation of miR-190 occurs at a transcriptional level, in a Sima-independent manner. Given that miR-190 is encoded in an intron of the *rhea* gene (S3 Fig), we investigated if *rhea* transcript levels are also upregulated in hypoxia. Similarly to miR-190, *rhea* was upregulated in hypoxia in a Sima-independent manner (Fig 7C). As a control of the effect of *sima* silencing, we assessed in the same embryos the expression of *fatiga B*, which is a well-known Sima target [30]. As shown in Fig 7D, *fatiga B* transcript levels were strongly increased in hypoxic wild type embryos, and this induction was reduced upon *sima* knock-down. Taken together, this set of experiments suggests that miR-190 is transcriptionally induced in hypoxia, as part of the *rhea* transcript, in a Sima-independent manner (S3 Fig).

## Discussion

*Drosophila melanogaster* has proved to be a useful model for studying the function of miRNAs as regulators of developmental programs, as well as in the maintenance of cellular homeostasis [38]. In the current work, we have carried out an *in vivo* screen, aimed at the identification of miRNAs involved in HIF-dependent hypoxic responses in *Drosophila*. Among 93 miRNAs tested, we identified miR-190, miR-274, miR-280 and miR-985 as positive regulators of Sima-dependent transcription. In mammalian cells, several miRNAs have been reported to participate in the response to hypoxia. Certain miRNAs, such as miR-20b, miR-199a, miR-155, miR-122, miR195, miR-335, miR-33a and miR-18a inhibit HIFα expression directly by binding its 3’UTR [45–52]. Other miRNAs, such as miR-424, miR-184, miR-210, miR-130, miR-494, miR-21 and miR-17 regulate HIFα expression positively through indirect mechanisms [53–60], which involve inhibition of negative regulators of this transcription factor. For example, miR-424 directly targets and reduces the expression of *cullin2* (*CUL2*), a scaffold component of the ubiquitin ligase complex that targets HIFα for degradation in the 26S proteasome [53]. Likewise, miR-184 inhibits another cardinal regulator of HIFα: the *factor inhibiting HIF-1* (*FIH-1*), an asparagine hydroxylase that hydroxylates HIFα, thereby inhibiting its association with the p300 transcriptional coactivator [54,61]. Another interesting example is the direct silencing of the *succinate dehydrogenase complex subunit D* (*SDHD*) by miR-210: inhibition of SDHD leads to accumulation of its substrate, succinate, which is in turn a product of HIFα prolyl hydroxylase (PHD) activity with inhibitory effects on the enzyme [62], which finally results in HIFαstabilization [55].

**Fig 8:**
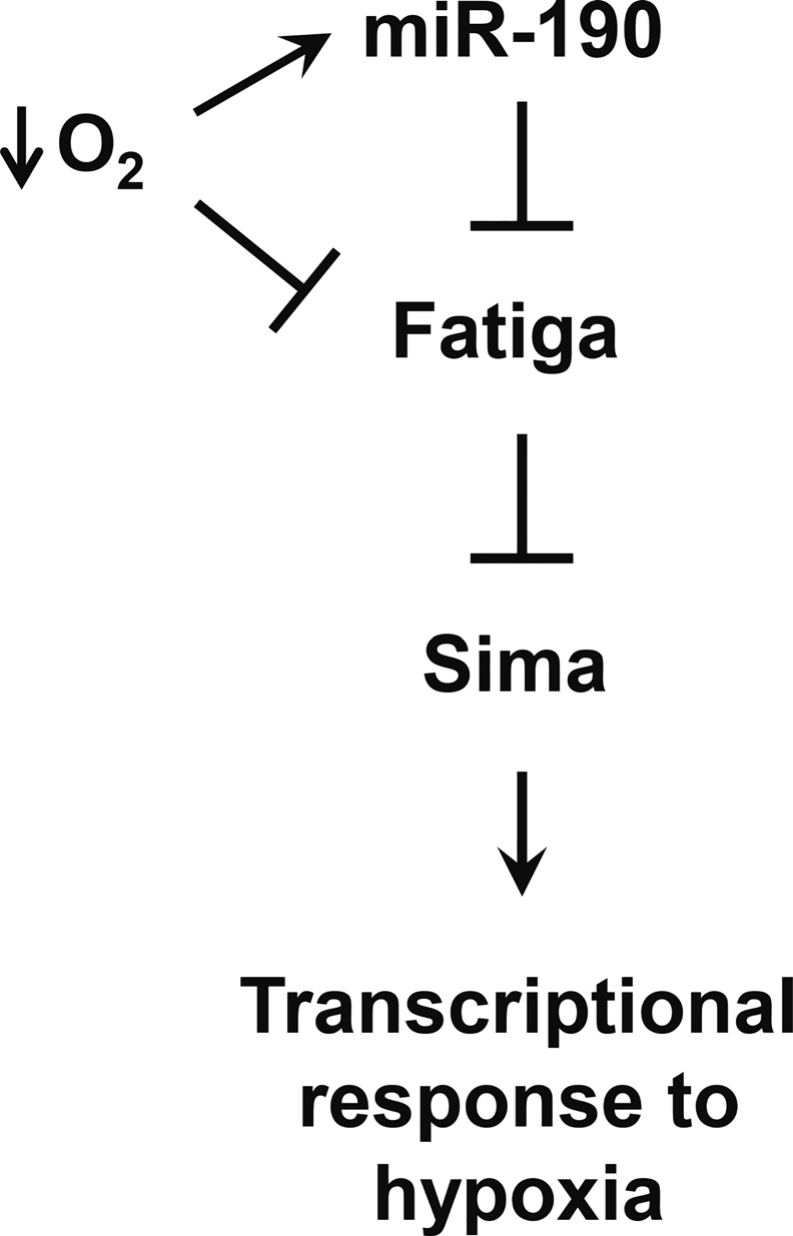
Model for the regulation of the hypoxic response by miR-190. miR-190 is a positive regulator of the hypoxic response by targeting directly *fatiga*, which is in turn the principal negative regulator of Sima and the response to hypoxia. Under low oxygen levels Fatiga activity is reduced, while miR-190 is upregulated.

In this study, we have shown that miR-190 directly targets and downregulates the oxygen sensor *fatiga*, thereby exerting positive regulation on the hypoxia master transcription factor Sima (Fig 8). miR-190 is induced in hypoxia, a condition in which Fatiga activity is also inhibited due to low oxygen availability (Fig 8), providing a mechanism by which miR-190 enhances the strength of the hypoxic response. To our knowledge, this is the first report of a miRNA that directly downregulates an oxygen sensing prolyl-4-hydroxylase.

As documented in the miRNA database miRBase (www.mirbase.org), miR-190 is broadly conserved in evolution, not only within the *Drosophilid* lineage [34], but also in distant taxa, including mammals. In most mammalian species, two miR-190 family members occur, miR-190a and miR-190b. The miR-190a locus lies in an intron of *talin2* (*TLN2*), which encodes a high molecular weight cytoskeletal protein. Remarkably, *Drosophila melanogaster* miR-190 is encoded in an intron of the gene *rhea* (S3 Fig), the homolog of *talin2 (TLN2)*. Intron 53 of human *TLN2-001* (which is 12,893 nucleotides long) and intron 14 of *rhea-RB* (which is 356 nucleotides long) only share sequence similarity within the miR-190 locus [33,34,63–70], reflecting the physiological relevance of this miRNA and perhaps some biological link with Rhea/Talin2. Interestingly, human PHD3 (also known as EGLN3), which is one of the three mammalian homologs of *Drosophila* Fatiga [19], has a predicted binding site for miR-190a, according to the miRNA target prediction databases TargetScan (www.targetscan.org) [71] and miRDB (mirdb.org) [72], even though with a relatively low score in both cases. Thus, it is possible that miR-190-dependent regulation of HIF-prolyl hydroxylases is conserved in evolution.

We found that *Drosophila* miR-190 is induced in hypoxia. Interestingly, mammalian miR-190 is upregulated in different types of cancer, including hepatocellular carcinoma [73,74], primary myelofibrosis [75], pancreatic [76], breast [77–79], rectal [80] and papillary thyroid cancer [81]. Hypoxic microenvironment is a common feature of many solid tumors [82,83], and most primary human cancers and their metastases exhibit increased levels of HIFα [84]. In addition to intratumoral hypoxia, genetic and epigenetic alterations can also stimulate HIF activity within tumors [82,84,85]. HIF promotes angiogenesis [82,86], metabolic switches [87], metastasis [88] and chemo/radio-resistance of cancer cells [89,90], and high levels of HIF are associated with poor patient prognosis and increased mortality [84,91]. On the other hand, many different miRNAs have been shown to play pivotal roles in cancer development, functioning as oncogenes or tumor suppressors [92–94]. Given that miR-190 is upregulated in diverse cancer types, our findings open the possibility that miR-190 contributes to HIFα stabilization in cancer cells, thereby enhancing tumor progression.

In line with this possibility, miR-190 directly inhibits the *PH domain leucine-rich repeat protein phosphatase* (*PHLPP*), a tumor suppressor protein that inactivates the kinase AKT through Ser437 dephosphorylation [95–97]. In human bronchial epithelial cells, trivalent arsenic (A^3+^) induces the expression of miR-190, which binds the 3’UTR of *PHLPP* transcript, decreasing PHLPP protein levels [95,96]. As a consequence, AKT phosphorylation and activation increase, finally resulting in vascular endothelial growth factor (VEGF) expression [95], which is induced following AKT activation [98]. Another *bona fide* miR-190 target is *IGF-1*, which is significantly reduced in serum of patients with hepatocellular carcinoma. Accordingly, miR-190b is upregulated in tumor tissues, contributing to insulin resistance through downregulation of IGF-1, which is associated with poor prognosis [73]. Thus, miR-190 favors carcinogenesis through distinct pathways.

Importantly, strengthening the notion of a possible involvement of miR-190 in mammalian responses to low oxygen, miR-190 is induced by hypoxia in a rat model of hypoxic pulmonary artery hypertension (PAH) [99–102]. miR-190 directly targets and represses the expression of *Kcnq5*, a member of the voltage-gated K^+^ channel family, resulting in augmented vasoconstriction of the pulmonary artery, a hallmark of hypoxic PAH [101].

In summary, the results reported here increase our understanding of the network controlling HIF-dependent responses to hypoxia, and open the possibility of analyzing the regulation exerted by additional miRNAs which may be part of this complex network.

## Materials and Methods

### Fly stocks

The UAS-miRNA fly collection utilized in this study was previously described [103]. The following fly stocks were from the Bloomington *Drosophila* Stock Center (Indiana University, Bloomington, IN, USA): *w*^*1118*^, *breathless*-Gal4, *engrailed*-Gal4, *dSRF*-Gal4, *actin*-Gal4, UAS-GFP, UAS-*white* RNAi and *miR-190*^*KO*^. The following stocks were from the Vienna *Drosophila* RNAi Center: UAS-*fatiga* RNAi (VDRC 103382), UAS-*sima* RNAi (VDRC 106504). The HRE-LacZ reporter [27], UAS-Fatiga B [30] and *fga*^*9*^*/*TM3 [29] lines were generated in our laboratory and previously described. The *rhea*^*79a*^ [40] mutant was kindly provided by Nicholas Brown.

### Hypoxic treatment and synchronized collection of embryos

Hypoxia was applied in a Forma Scientific 3131 incubator, by regulating the proportions of oxygen and nitrogen. To obtain synchronized individuals, embryos were collected on egg-laying agar plates for 4 h, and then incubated at 18°C or 25°C in normoxia until the desired stage. When necessary, embryos or first-instar larvae were sorted to obtain the desired genotypes using a fluorescent Olympus stereomicroscope MVX10.

### X-gal staining

For X-gal stainings, embryos were dechorionated in bleach for 1 min, incubated with heptane for 5 min, fixed with glutaraldehyde 0.5% in PBS for 20 min and then washed three times for 5 min in PT 0.3% (PBS containing 0.3% Triton-X 100). Samples were incubated 1 h with the staining solution (5 mM K4Fe(CN)6, 5 mM K3Fe(CN)6, 0.2% X-gal) at 37°C. After three washes with PT 0.3%, samples were analyzed using an Olympus stereomicroscope MVX10; and photographed after mounting in glycerol 80% with an Olympus BX60 microscope equipped with an Olympus DP71 digital camera.

### miRNA overexpression screen

The screen was performed using the Fly Condo (Flystuff, San Diego, CA, USA), which contains 24 independent chambers, allowing for high-throughput collection of *Drosophila* embryos. In each chamber, we placed adult males bearing the *btl*-Gal4 driver and the HRE-LacZ reporter, together with females of one miRNA line or a wild type line (w^1118^) as a negative control. Embryos from the offspring were collected in the 24-well stainless steels mesh plate provided with the condo and subjected to hypoxia (11% O_2_), for 4 h. Next, we evaluated the expression of the HRE-LacZ reporter performing X-gal stainings of the embryos within the mesh plate.

### Quantification of tracheal phenotypes

First-instar larvae were placed in fresh vials, at a density of 20 individuals per vial. When they reached the third-instar wandering stage, larvae were anesthetized with ether and ramifications of the terminal cell of the trachea in the dorsal branch of the third segment were counted and photographed using bright-field microscopy.

### Extraction of total RNA and quantitative real time PCR

Embryos were incubated under hypoxia (11%, 8% or 5% O_2_) or normoxia for 4 h, at 25°C. Next, total RNA was isolated using Trizol reagent (Invitrogen, Carlsbad, CA, USA) from embryos of stages 14–17. Genomic DNA was removed from RNA samples using Ambion’s DNase (Ambion, Austin, TX, USA). Samples (1 μg) were reverse-transcribed with the M-MLV Reverse Transcriptase (Invitrogen, Carlsbad, CA, USA), following the manufacturer´s instructions, using oligo-dT as a primer. The concentration and integrity of RNA and cDNA were determined using Nanodrop ND-1000 spectrophotometry and gel electrophoresis.

The resulting cDNA was used for quantitative real time PCR, using a MX3005P instrument (Stratagene, La Jolla, CA, USA). The real time PCR reaction contained: 1 μL Sybr Green 1/1000, 0.3 μL ROX reference dye 1/10 (Invitrogen, Carlsbad, CA, USA), 0.2 μL of Taq DNA Polymerase Recombinant (Invitrogen, Carlsbad, CA, USA), 2.5 μL Buffer 10X, 1 μL MgCl_2_ 50 mM, 0.5 μL dNTP mixture 10 mM (Invitrogen, Carlsbad, CA, USA), 1 μL of sense primer 10 μM, 1 μL of anti-sense primer 10 μM, 5 μL of template cDNA 1/30, 4.2 μL glycerol 30% and 8.3 μL of H_2_O. The thermal cycling conditions were the following: 95°C for 10 min, followed by 40 cycles at 95°C for 30 s, 60°C for 1 min and 72°C for 1 min, finishing with a cycle for the melting curve of 95°C for 1 min, 60°C for 30 s and 95°C for 30 s. Relative mRNA expression was normalized using *rpl29*, *rpl32* or *GAPDH* as internal controls.

For quantification of miR-190 levels, the NCode VILO miRNA cDNA Synthesis Kit (Invitrogen, Carlsbad, CA, USA) was used, following manufacturer´s instructions. The 2S rRNA was used for normalization in quantitative real time PCR determinations of miR-190.

### Immunostaining

Larvae were dissected in PBS, fixed in 4% formaldehyde (Sigma, St. Louis, MO, USA) for 40 min at room temperature and then washed three times for 10 minutes in PT 0.3% (PBS containing 0.3% Triton-X 100). Samples were blocked with bovine serum albumin 5% in PT 0.3% (PBT) for 2 h and then incubated with the primary antibody in PBT overnight at 4°C. After washing three times for 15 min with PT 0.3%, tissues were incubated for 2 h at room temperature with the secondary antibody in normal goat serum 5% diluted in PT 0.3%. Next, samples were washed, imaginal discs were separated and mounted in glycerol 80%. Images were analyzed and captured using a Carl Zeiss LSM510 Meta Confocal Microscope.

We used mouse anti-Engrailed (1:100; Developmental Studies Hybridoma Bank, Iowa, IA, USA) primary antibody and donkey anti-mouse Cy5 (Jackson, ImmunoResearch Laboratories Inc., West Grove, PA, USA) secondary antibody. Fluorescence of GFP and RFP was analyzed without antibody staining.

### Plasmids

The copper-inducible pMT/V5-His plasmid (Invitrogen, Carlsbad, CA, USA) was utilized as a backbone vector for generating reporter constructs. For generation of pMT-Luciferase renilla (pMT-Renilla), the coding sequence of renilla luciferase was subcloned from a pRL-SV40 vector (Promega, Madison, WI, USA) into HindIII/XbaI sites of pMT/V5-His. For the pMT-Luciferase firefly reporter construct (pMT-Firefly), the firefly luciferase coding sequence was subcloned from pGL3 vector (Promega, Madison, WI, USA) into EcoRI/XbaI sites of pMT/V5-His. *fatiga* 3’UTR sequence was generated by PCR from cDNA prepared from *Drosophila yellow white* embryos and cloned into XbaI/ApaI restriction sites of the pMT-Firefly plasmid. The primers utilized were:

Forward (Fw): 5’-GCTCTAGACCCAAGCCGACAGCGCAGCT-3’;

Reverse (Rv): 5’-GCCATTGGGCCCCATCAGCTCAGGCTTTTGTTTA-3’.

Point mutations in miR-190 binding site at the *fatiga* 3’UTR were introduced by nested PCR with the following primers:

Fw: 5’-CTGTAAATCATGAAGTATGTATATTTATGCCCTCGCTACATATTGTATG-3’;

Rv: 5’-CATACAATATGTAGCGAGGGCATAAATATACATACTTCATGATTTACAG-3’.

The Luc-*CG10011* 3’UTR reporter and pAc-miR-12 were a gift from E. Izaurralde [44]. The pAc-miR-190 overexpression plasmid was kindly provided by M. Milán [104]. The pAc-5.1/V5-His (Invitrogen, Carlsbad, CA, USA) was used as a negative control.

### Cell cultures, transfections and luciferase assays

Semi-adherent Schneider (S2R+) *Drosophila* cells were maintained in Schneider *Drosophila* medium (Sigma, St. Louis, MO, USA) supplemented with Penicillin (50 U/ml, Invitrogen), Streptomycin (50 μg/ml, Invitrogen) and 10% fetal bovine serum (Invitrogen, Carlsbad, CA, USA) at 25°C in 25 cm^2^ T-flasks.

Cells were seeded in 24-well plates at a 35000 cells per well density and 0.3 μg of total DNA was transfected employing the Effectene transfection reagent (Qiagen, Valencia, CA, USA). All pMT-Firefly-3’UTR constructs were co-transfected at a 1:1 proportion with pMT-Renilla to normalize transfection efficiency. Expression of luciferase from pMT vectors was induced 24 h after transfection by addition of 0.7 mM CuSO_4_ for 7 h. Firefly and renilla luciferase activities were measured by the Dual-Glo Luciferase Assay System (Promega, Madison, WI, USA), following the instructions of the manufacturer, in a Veritas Microplate Luminometer (Turner BioSystems).

### Statistical analysis

Data are expressed as mean ± standard deviation (SD). Infostat Statistical Software was used for statistical analysis. Comparisons were performed using one- or two-way analysis of variance (ANOVA) followed by Fisher's protected least significant difference (LSD) as *post hoc* test, or unpaired two-tailed Student's *t*-test. Data were tested for normality (Shapiro–Wilks test) and variance homogeneity (Levene test) to use parametric statistical analysis. If data did not fulfill these statistical criteria, Welch's correction or the Kruskal-Wallis one-way ANOVA non-parametric test were used. A p<0.05 was considered statistically significant.

## Supporting Information

S1 Table List of miRNAs evaluated in the screen. A complete list of all UAS-miRNAs tested in the screen is presented.
(XLSX)

S1 Fig Expression of *sima* RNAi is effective in reducing *sima* mRNA levels and provokes lethality in individuals exposed to hypoxia. **(A)** Normoxic third instar larvae in which ubiquitous expression of *sima* RNAi was induced with an *actin*-Gal4 driver downregulated *sima* mRNA levels to 32% of their control siblings bearing the *act*-Gal4 driver only **p<0.01; unpaired two-tailed Student’s *t*-test. Error bars represent SD; n ≥ 3 per group. **(B)** *sima* silencing provoked lethality in larvae exposed to hypoxia. First instar larvae developed in normoxia that expressed *sima* RNAi were transferred to an incubator with 5% O_2,_ and the number of larvae undergoing pupariation was recorded 7 days later in comparison with that of siblings exposed to the same treatment but carrying the *act*-Gal4 driver only. Error bars represent SD; n ≥ 20 larvae per group.
(TIF)

S2 Fig miR-190 overexpression enhances induction of Sima endogenous target genes in cell culture in normoxia. miR-190 was overexpressed in normoxic *Drosophila* S2R+ cells by transfection with 300 ng of a pAc-miR-190 plasmid or an empty vector as a control. Analysis by real time RT-PCR revealed that overexpression of the miRNA provoked upregulation of the endogenous Sima target genes *fatiga B* (*fgaB*) and *heat shock factor* (*hsf*). **p<0.01, *p<0.05; unpaired two-tailed Student’s *t*-test (in FgaB quantitative RT-PCR, data were transformed using the reciprocal number to fulfill variance homogeneity criteria). Error bars represent SD; n ≥ 3 per group.
(TIF)

S3 Fig Schematic representation of the *rhea* locus including one of its introns where miR-190 is encoded. Structure of the *rhea-RB* primary transcript is shown, along with those of transcripts of the two neighboring loci *ergic-53-RB* and *CG6638-RA*. Grey boxes represent coding exons, white boxes non-coding exons and lines introns. The region encompassing exon 14 to exon 16 of *rhea-RB* is amplified to show that miR-190 (red) is encoded within its intron 14. The *rhea*^*79a*^ deletion (shown in the upper part of the scheme) covers the *ergic-53*, *rhea and CG6638* loci.
(TIF)

S4 Fig Overexpression of miR-190 does not affect *sima* mRNA levels. Embryos ubiquitously overexpressing miR-190 under the control of an *actin*-Gal4 driver, were either kept in normoxia or exposed to mild hypoxia (11% O_2_) for 4 h. miR-190 overexpression did not affect *sima* transcript levels as compared to control embryos bearing the *act*-Gal4 driver only, as assessed by real time RT-PCR. Error bars represent SD; n ≥ 3 per group.
(TIF)

S5 Fig miR-12-dependent downregulation of the *CG10011* 3’UTR reporter. *Drosophila* S2R+ cells were co-transfected with the *CG10011* 3’UTR reporter along with a pAc-miR-12 overexpression plasmid, or an empty vector as a control. Another plasmid encoding *Renilla* luciferase was co-transfected for normalization. As expected, luciferase expression was strongly reduced in cells transfected with the miR-12 overexpression plasmid. *p<0.05; unpaired two-tailed Student’s *t*-test. Error bars represent SD; n ≥ 3 per group.
(TIF)

S1 Dataset Data collection of all the figures. (XLSX)
