# Supplementary figures and images for: miR-190 Enhances HIF-Dependent Responses to Hypoxia in Drosophila by Inhibiting the Prolyl-4-hydroxylase Fatiga
Source: PLoS Genet. 2016 May 25;12(5):e1006073. doi: 10.1371/journal.pgen.1006073 (PMC4880290; doi:10.1371/journal.pgen.1006073)

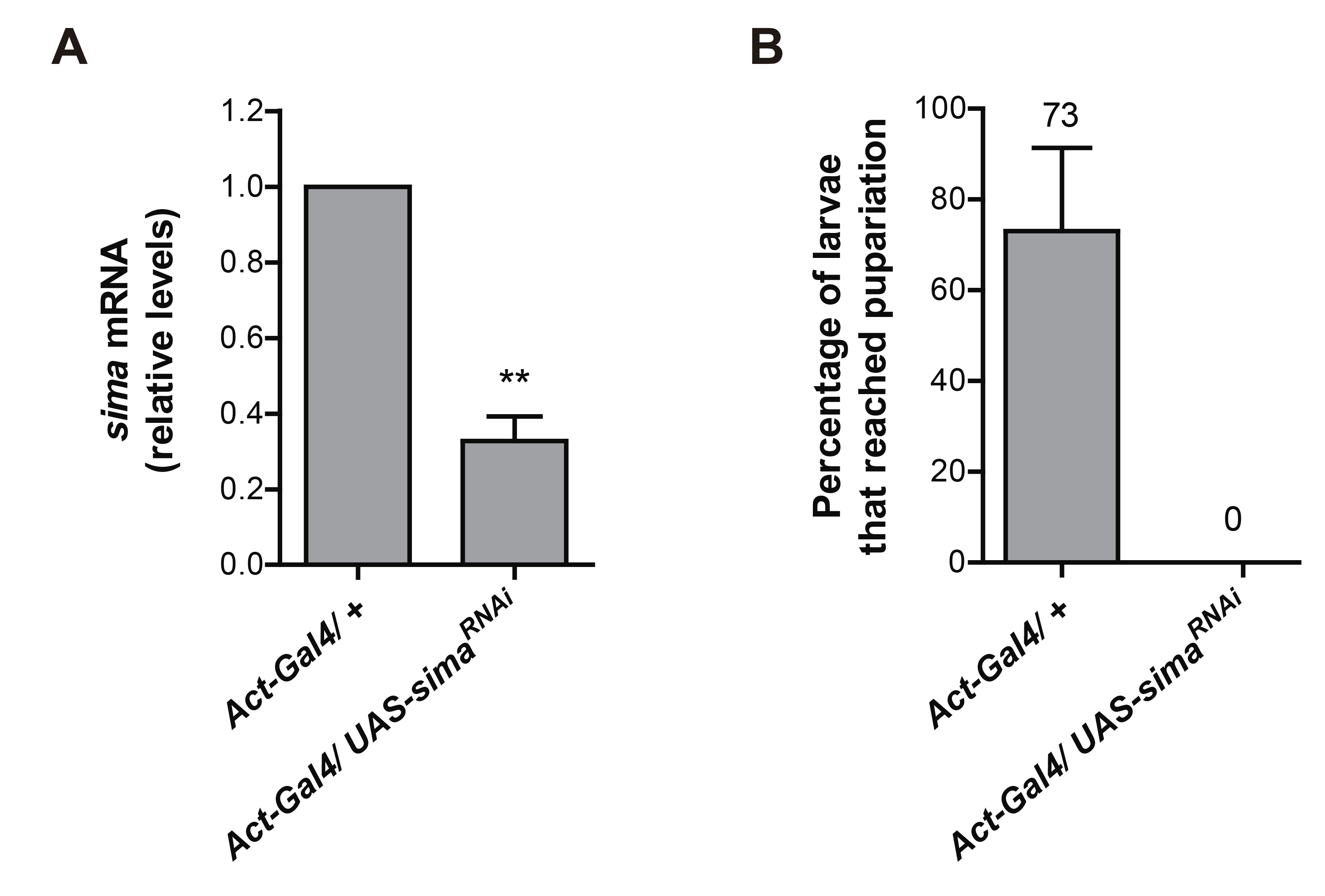

Supplement: S1 Fig — (A) Normoxic third instar larvae in which ubiquitous expression of sima RNAi was induced with an actin-Gal4 driver downregulated sima mRNA levels to 32% of their control siblings bearing the act-Gal4 driver only **p<0.01; unpaired two-tailed Student’s t-test. Error bars represent SD; n ≥ 3 per group. (B) sima silencing provoked lethality in larvae exposed to hypoxia. First instar larvae developed in normoxia that expressed sima RNAi were transferred to an incubator with 5% O2, and the number of larvae undergoing pupariation was recorded 7 days later in comparison with that of siblings exposed to the same treatment but carrying the act-Gal4 driver only. Error bars represent SD; n ≥ 20 larvae per group. (TIF) [file pgen.1006073.s002.tif]

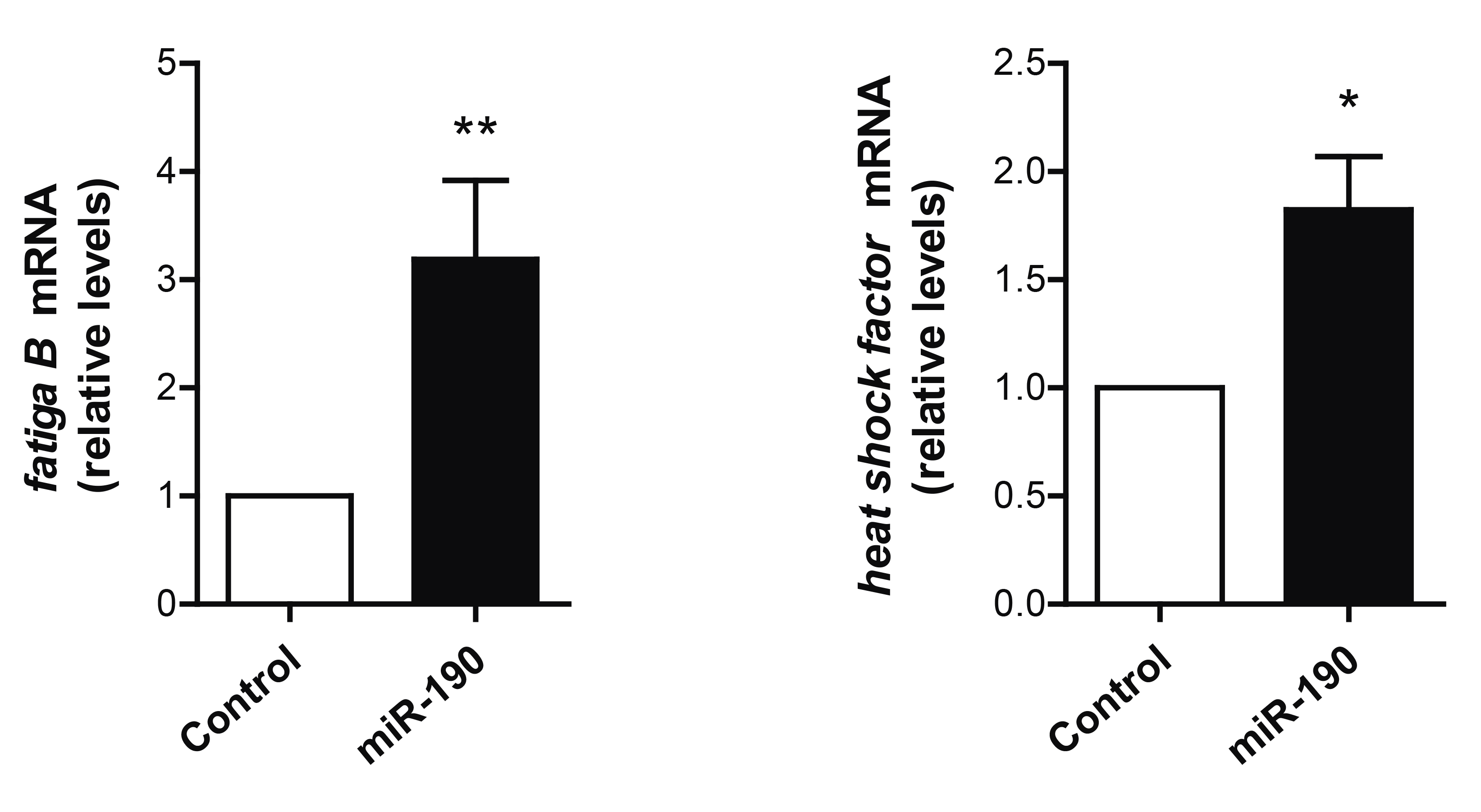

Supplement: S2 Fig — miR-190 was overexpressed in normoxic Drosophila S2R+ cells by transfection with 300 ng of a pAc-miR-190 plasmid or an empty vector as a control. Analysis by real time RT-PCR revealed that overexpression of the miRNA provoked upregulation of the endogenous Sima target genes fatiga B (fgaB) and heat shock factor (hsf). **p<0.01, *p<0.05; unpaired two-tailed Student’s t-test (in FgaB quantitative RT-PCR, data were transformed using the reciprocal number to fulfill variance homogeneity criteria). Error bars represent SD; n ≥ 3 per group. (TIF) [file pgen.1006073.s003.tif]

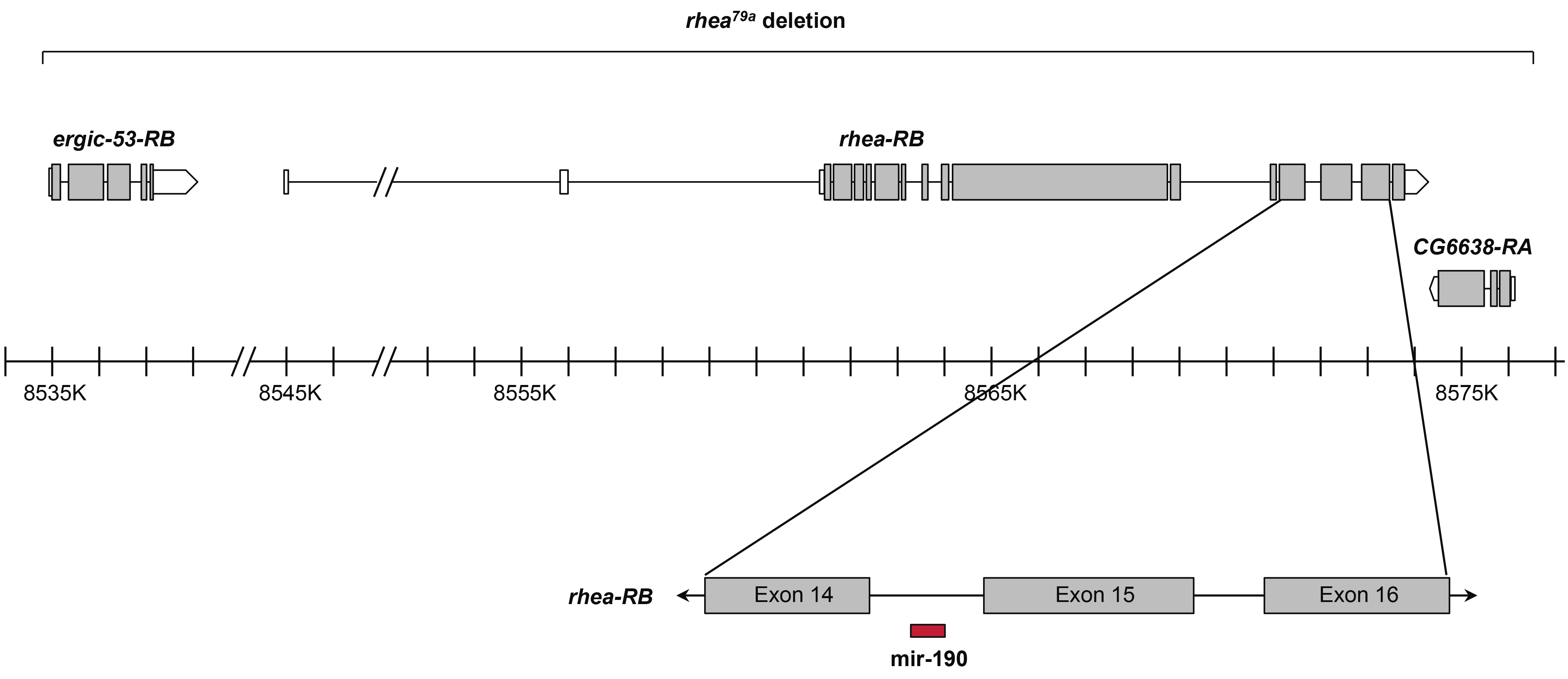

Supplement: S3 Fig — Structure of the rhea-RB primary transcript is shown, along with those of transcripts of the two neighboring loci ergic-53-RB and CG6638-RA. Grey boxes represent coding exons, white boxes non-coding exons and lines introns. The region encompassing exon 14 to exon 16 of rhea-RB is amplified to show that miR-190 (red) is encoded within its intron 14. The rhea79a deletion (shown in the upper part of the scheme) covers the ergic-53, rhea and CG6638 loci. (TIF) [file pgen.1006073.s004.tif]

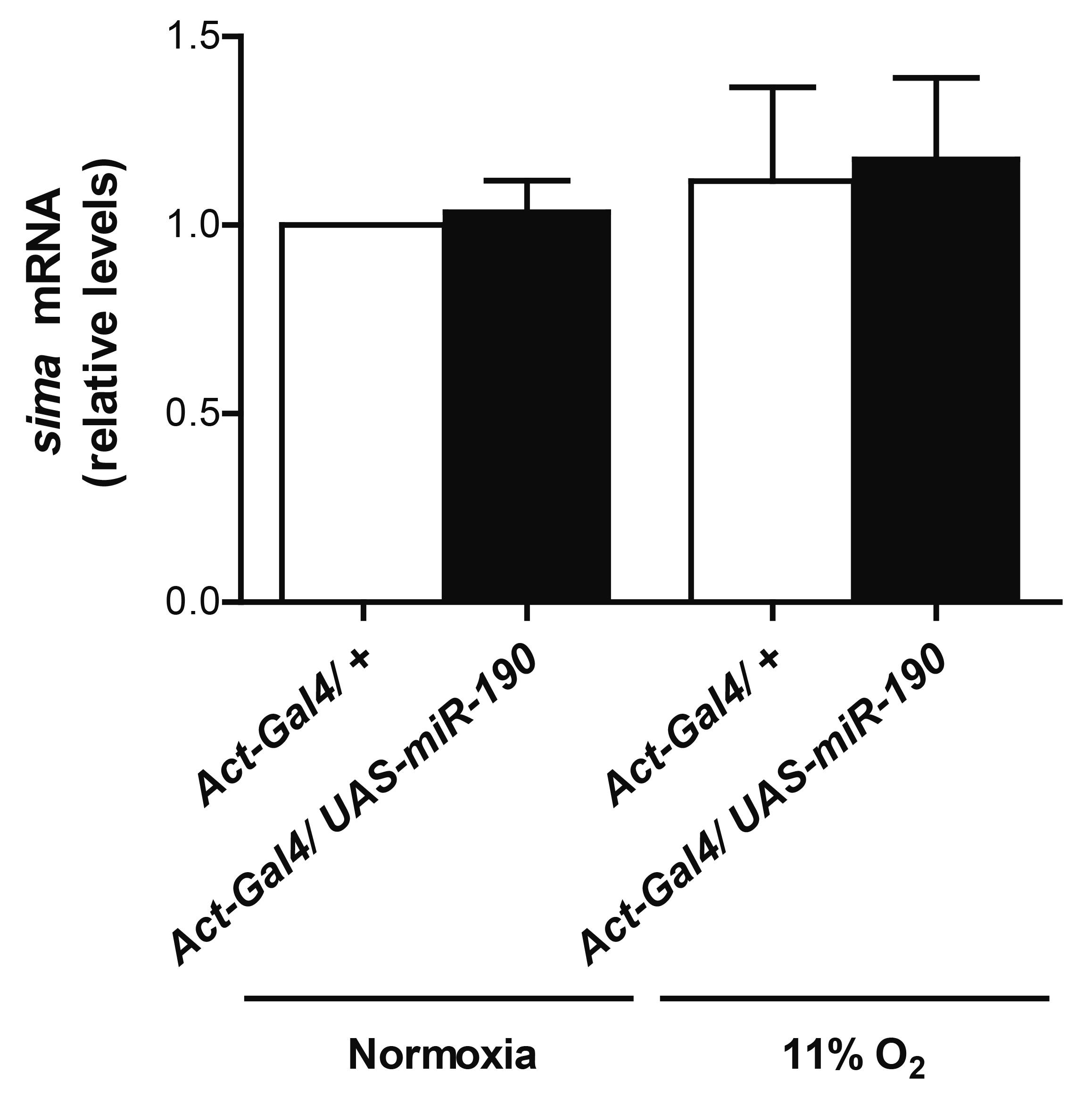

Supplement: S4 Fig — Embryos ubiquitously overexpressing miR-190 under the control of an actin-Gal4 driver, were either kept in normoxia or exposed to mild hypoxia (11% O2) for 4 h. miR-190 overexpression did not affect sima transcript levels as compared to control embryos bearing the act-Gal4 driver only, as assessed by real time RT-PCR. Error bars represent SD; n ≥ 3 per group. (TIF) [file pgen.1006073.s005.tif]

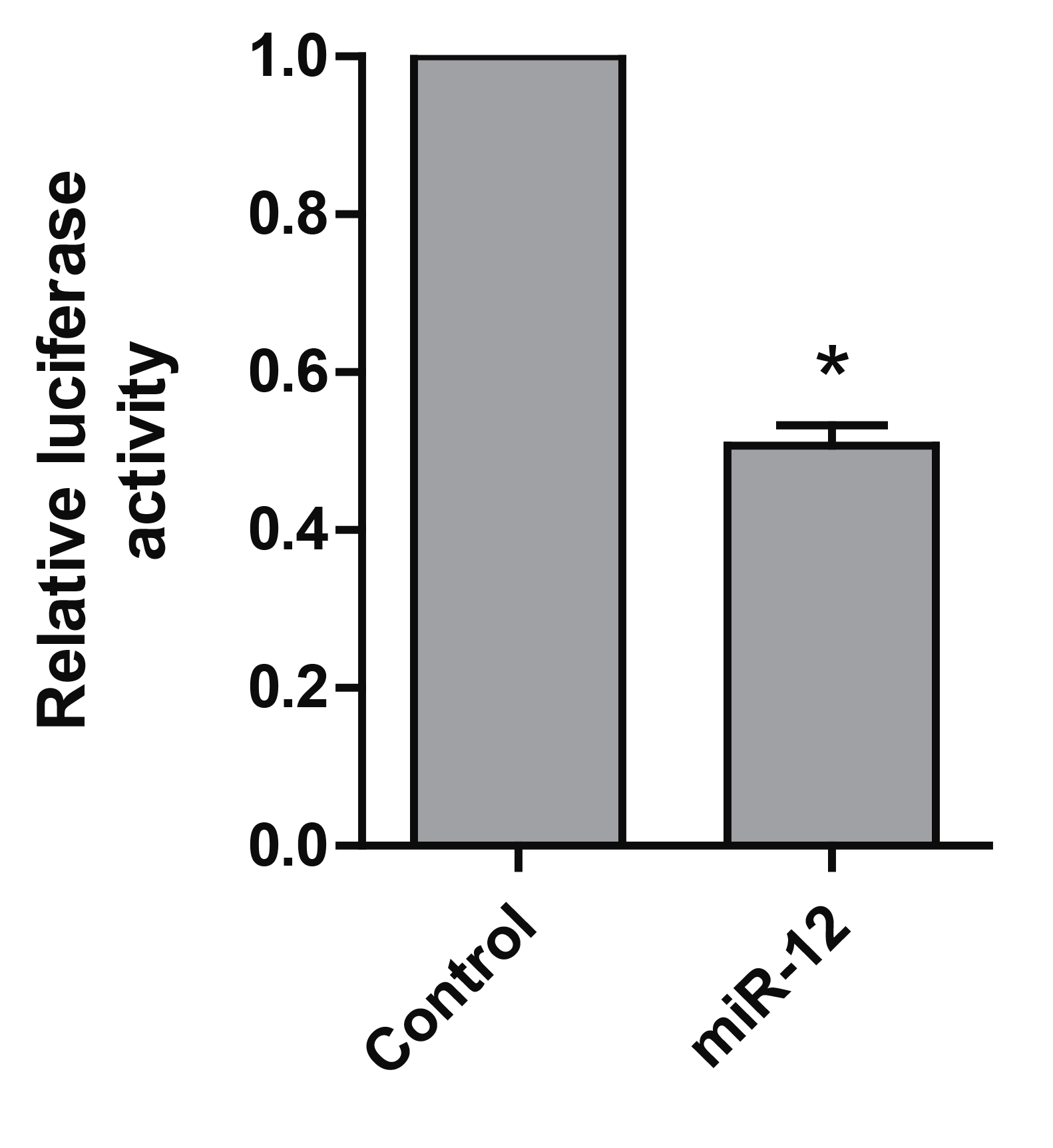

Supplement: S5 Fig — Drosophila S2R+ cells were co-transfected with the CG10011 3’UTR reporter along with a pAc-miR-12 overexpression plasmid, or an empty vector as a control. Another plasmid encoding Renilla luciferase was co-transfected for normalization. As expected, luciferase expression was strongly reduced in cells transfected with the miR-12 overexpression plasmid. *p<0.05; unpaired two-tailed Student’s t-test. Error bars represent SD; n ≥ 3 per group. (TIF) [file pgen.1006073.s006.tif]
